# An analysis of ambulance re-contacts after non-conveyance: a retrospective cohort study in the Netherlands

**DOI:** 10.1186/s13049-025-01332-3

**Published:** 2025-02-01

**Authors:** Susanne E. de Loor, Tessa Verheij, Thomas Karol, Franciscus G. M. H. M. Cuppen, Frits van Dijk, Femke Goldstein, Joyce Janssen, Remco H. A. Ebben

**Affiliations:** Emergency Medical Service, Public Health and Safety Region Gelderland-Midden, Arnhem, The Netherlands

**Keywords:** Non-conveyance, Emergency medical services [MeSH], Patient safety [MeSH]

## Abstract

**Background:**

Non-conveyance is an increasing part of ambulance care and has to be safe. One of the indicators to measure safety is an ambulance re-contact within 72 h. However, solely measuring the percentage of re-contacts has limited validity as it lacks insight in actual reasons of an ambulance re-contact. Therefore, the aim of our study was to analyze the incidence, reasons and outcomes of ambulance re-contacts within 72 h after non-conveyance.

**Methods:**

We conducted a one year (2022) retrospective study in one EMS region in the Netherlands. Medical records of all non-conveyance runs with a re-contact were analyzed using a framework to categorize re-contact reasons in illness-related, patient-related, professional-related, and unrelated. Re-contact outcomes were measured in terms of (non-)conveyance and mortality.

**Results:**

585/13.879 (4.2%) non-conveyance runs had a re-contact within 72 h. 547/585 (93.5%) re-contacts could be categorized with the framework. Re-contacts were related to the illness (n = 267, 48.8%), the patient (n = 130, 23.8%), the professional (n = 106, 19.4%) and unrelated (n = 44, 8.0%). Four subreasons accounted for 68.5% of reasons for re-contacts: progression of disease (19.4%), recurrent disease process/exacerbation (18.6%), reassessment and ambulance request by another medical professional (15.9%), and psychiatric disorder and/or substance abuse (14.6%). 403/547 (73.7%) patients with a re-contact were conveyed to the hospital. Mortality rate for patients with a re-contact was 0.5%.

**Conclusions:**

Re-contact incidence after non-conveyance is relatively low, with a very small part of re-contacts related to ambulance care professionals making errors in diagnosis or treatment. Combined with low re-contact mortality, this indicates safe non-conveyance decisions. Re-contacts as quality indicator cover a variety of reasons, with almost half of the re-contacts being related to illness. Four subcategories accounted for the majority of all reasons for re-contacts: progression of disease, recurrent disease process/exacerbation, reassessment and ambulance request by another medical professional, and psychiatric disorder and/or substance abuse. Three-quarters of the patients were conveyed, although more re-contacts due to patient related reasons ended in non-conveyance again.

## Background

In recent years, there is a growing demand for care from emergency medical services (EMS) with an increasing number of ambulance deployments [1]. Patients are aging, have co- and multimorbidity’s, have to live at home for a longer time with poor health literacy, and have limited access to primary care but easy access to emergency care [2–4]. Besides an increasing burden on EMS capacity the increasing EMS patient flow contributes to emergency department (ED) overcrowding [5].

Within this increase in volume and complexity, not all patients to whom an ambulance is dispatched are conveyed to the hospital. The patient can also be left at home, whereby the patient receives on-site diagnostics, treatment, (self-care) instructions and possible referral to the General Practitioner (GP) or other healthcare provider. This phenomenon is called non-conveyance and is defined as an appropriate ambulance deployment where the patient after on-scene assessment and/or treatment does not require conveyance with medical personnel and equipment to a healthcare facility [6]. Non-conveyance can be initiated by the professional, the patient, or through shared decision making.

Non-conveyance has become a substantial part of ambulance care, as nationally and internationally there has been an increase in the number of non-conveyances in recent years. In the Netherlands 24% of all ambulance runs ended up in a non-conveyance in 2022 [7]. A systematic review from 2017 reported non-conveyance rates for general patient populations ranging from 3.7 to 93.7% [8]. More recent studies have shown similar results, with non-conveyance percentages varying from 13.8 to 29.6% [2, 9]. When a patient is not conveyed in the Dutch healthcare system, the patient receives instructions on self-care, when to seek additional care from a GP, and when to re-contact an ambulance. Also, patients might present themselves at an ED. However, there is no option to contact private hospitals/private EMSs. Literature shows that 13.0% of the patients visit their own GP within 24 h after being non-conveyed, and a small proportion attends the ED [8].

Non-conveyance research has primarily focused on characteristics of patients and ambulance runs, outcomes and safety. To measure the quality and safety of non-conveyance, a variety of indicators is used, including mortality, ED attendance, hospital admission, and ambulance re-contacts [10]. These indicators lie within the EMS system or within the chain of emergency care. The most common used quality indicator within the EMS system is ambulance re-contacts, with the most common time-interval of 48 h [10]. Literature shows time-intervals from 24 h to 7 days and reported re-contact rates of 6.1% (24 h), 2.3–2.5% (48 h) and 7.4–13.5% (7 days) [8]. Although the indicator 'ambulance re-contact' gives an impression of the quality of non-conveyance, the indicator has limited validity. These limitations arise from the lack of the degree of relationship between the initial consultation and the re-contact [11]. The reasons for a re-contact are often unclear and have not been investigated in the scientific literature. This means that there is a lack of in-depth insight and subsequently a qualitative interpretation of the quantitative indicator. Therefore, the aim of our study was to analyze the incidence, reasons and outcomes of ambulance re-contacts within 72 h after non-conveyance.

## Methods

### Design

The study had a retrospective, descriptive design. The study is reported conform the STROBE-statement [12].

### Setting and population

Ambulance care in the Netherlands is provided by 25 regional EMSs with 1.489.572 ambulance runs in 2022, of which 388.797 ambulance runs ended in non-conveyance [7]. This study took place in EMS region Gelderland-Midden which is located in the eastern part of the Netherlands and provides ambulance care for a 700.000 population. In 2022 this EMS performed 50.924 ambulance runs, of which 13.879 ended in non-conveyance [7]. Ambulances are dispatched by the regional emergency medical dispatch center using the Dutch Triage Standard. Ambulances are dispatched with urgency level A1 (arrival < 15 min), A2 (arrival < 30 min), and B (ordered ambulance transportation). Within this EMS ambulances are staffed by a driver accompanied with an ambulance professional being either an ambulance nurse or a bachelor of health (BH). Ambulance nurses are registered nurses who have followed several specialist educational programs in critical care, coronary care or ED care. After completing (one of) these programs, nurses enter an 8-month specific national EMS training course to become qualified as ambulance nurse (bachelor’s degree—NLQF-6) [13]. A bachelor of health (NLQF-6) followed a four-year educational program, with a specialization in emergency care (ambulance or ED), anesthesia or coronary care. After graduation the BH follows the same national EMS training course to become registered as an ambulance professional [14]. Ambulance professionals work autonomously with Dutch National Protocols [15]. The National Protocols are updated regularly and cover all aspects of prehospital care. Ambulance professionals are allowed to make autonomous medical decisions based on these National Protocols, including decisions on non-conveyance. If necessary, ambulance professionals can consult EMS medical supervisors who have EMS medical responsibility, or a GP or Medical Specialist to discuss medical decisions and whether or not to convey the patient.

### Data collection

Each ambulance run is stored in an EMS database and has an unique identification number. For this study we selected ambulance runs from 2022 that had patient contact, ended in non-conveyance and had a re-contact within 72 h. For each ambulance run meeting inclusion criteria, we performed a medical record review. The following pre-structured variables were extracted from the medical record: sex, age, time of the day, day of the week, applicant, level of urgency (A1, A2 or B), medical specialism and diagnosis, and outcome (conveyance/non-conveyance and death). In addition to the pre-structured variables, we extracted free text fields from the medical record. In these free text fields, ambulance professionals register additional information about anamnesis, diagnosis, treatment and considerations. From these free text fields, diagnosis and reasons for the re-contact were extracted.

All included ambulance runs were analyzed using a framework to categorize re-contact reasons. As a framework for EMSs to analyze their re-contacts is lacking, we used a framework for ED-settings [16]. This framework describes 3 categories to analyze un-scheduled ED visits: illness-related, patient-related, and professional-related. Based on expert opinion, we added the main category ‘unrelated’ and added subcategories to make the framework more suitable for the ambulance setting. These experts were the medical supervisors of the EMS, being two emergency physicians. These supervisors are responsible for medical care and medical policy within the EMS. The framework is presented in Table 1.

**Table 1:** Main categories and subreasons for re-contacts

Main category re-contact	Sub reason re-contact
*Professional related*	*Definition*
Treatment error*	The ambulance professional made the right diagnosis during the initial non-conveyance contact, but made a treatment error
Misdiagnosis*	Medical record review reveals a diagnosis or problem missed by the ambulance professional who saw the patient on the initial non-conveyance contact
Reassessment and ambulance request by another medical/care professional**	The patient has an unchanged complaint (pattern) compared to the initial non-conveyance contact, but is reassessed by another professional (general practitioner/specialist/mental health care) who decides to request ambulance care
*Patient related*	
Refusal of medical care/treatment*	The patient refused medical care/treatment on the initial non-conveyance contact, this was against advice of the ambulance professional
Non-compliance with self-care instructions*	The patient did not comply with self-care instructions given on the initial non-conveyance contact
Psychiatric disorder and/or substance abuse*	The patient has a psychiatric disorder and/or uses drugs or alcohol, which causes him/her to repeatedly request ambulance care for the same or similar problems
Non-compliance with instructions to visit own general practitioner*	The patient was instructed to return to the GP for re-evaluation On the initial non-conveyance contact, but did not go
The patient and/or relatives are worried*	The patient’s and/or relatives worrying/anxiety caused him/her to seek ambulance care for the same or similar problem. During the re-contact no additional diagnostics were performed and medical management consisted of reassurance only
*Illness-related*	
Recurrent disease process/exacerbation*	The patient has a disease that tends to have recurrent exacerbations/episodes. The patient was treated appropriately during the initial non-conveyance contact, with resolution of symptoms, but later returned with a second exacerbation/episode of the disease
Complication*	The patient was treated appropriately during the initial non-conveyance contact, but seeked new ambulance care because of a complication of the disease or unpredictable side effect of treatment (e.g. allergic drug reaction)
Progression of disease*	During the initial non-conveyance contact the patient was treated appropriately and an adequate safety net/follow-up care was initiated by the ambulance professional. However, the patient's disease or problem got worse (no recurrent exacerbation/episode), and he/she seeked ambulance care
Additional diagnostics performed, no change in diagnosis*	The patient presented with the same or similar problem as during the initial non-conveyance contact, additional diagnostics were performed but there was no change in the initial diagnosis or treatment
Related disease**	During the re-contact a new disease/problem is present that was not present during the initial non-conveyance contact, but the diseases/problems have a clear relationship with each-other
*Unrelated*	
Unrelated disease**	During the re-contact a new disease/problem is present that was not present during the initial non-conveyance contact, but the diseases/problems have no clear relationship with each-other

*Based on literature Van der Linden et al. (2014)

**based on expert opinion

All runs were independently assessed and categorized by a pair of two assessors, using the framework. The assessors were five ambulance nurses (SL, TV, FD, FG, JJ) and one bachelor or health (TK), and were trained to use the framework. To increase the interrater reliability a calibration session was performed at the start of the study, where the first 10 ambulance runs were assessed by all assessors. The assessors received datasets that were anonymized for patient en professional characteristics. Assessor pairs were blinded for each other’s categorization. The data of the two independent assessors were combined by RE. When both categorizations matched, the final category was assigned. In case of disagreement a third assessor was involved for a final decision.

### Statistical analysis

Based on the descriptive statistics, the incidences and outcomes for re-contacts in 24, 48 and 72 h were calculated. To compare variables between the main categories, Chi-square test, Cramer’s’ V and Kruskal–Wallis test were performed. Statistical significance was set at *p* = value < 0.05. Results are presented in frequencies and cross-tabulation tables. All data were analyzed with SPSS version 28.

## Results

### Incidence

In 2022 there were 13.879 non-conveyance runs, with 585 (4.2%) re-contacts within 72 h. The incidence rates for the 0 h-24 h, 24 h-48 h and 48 h-72 h timeframes were 2.8% (n = 392), 0.9% (n = 125) and 0.5% (n = 68). 392/585 (67.0%) of the re-contacts took place within 24 h after the initial non-conveyance run, 125/585 (21.4%) between 1 and 2 days and 68/585 (11.6%) between 2 and 3 days.

### Main reasons for re-contacts

Of the 585 re-contacts, 547 (93.5%) could be categorized within the framework (Table 2). 38/585 (6.5%) could not be categorized due to missing data/poor registration. Re-contacts were related to illness (48.8%), the patient (23.8%), the professional (19.4%) or were unrelated (8.0%). Illness related re-contacts were the most common reason in all timeframes. Professional related re-contacts occurred more often during the first 24 h after non-conveyance, in comparison to re-contacts after 24 h. Unrelated re-contact occurred more often in the 48 h-72 h timeframe. χ^2^ showed a significant but small association between the main categories and timeframes (Cramer’s V 0,122, *p* = 0.012).

**Table 2:** Characteristics re-contacts and main categories (n = 547)

Variable	Illness related (n = 267) N (%)	Patient related (n = 130) N (%)	Professional related (n = 106) N (%)	Unrelated (n = 44) N (%)	Total N (%)	Statistics
*Timeframe*						*p* = .012*
0 h–24 h	177 (48.2)	81 (22.1)	86 (23.4)	23 (6.3)	367 (67.1)	
24 h–48 h	58 (49.6)	33 (28.2)	14 (12.0)	12 (10.3)	117 (21.4)	
48 h–72 h	32 (50.8)	16 (25.4)	14 (12.0)	9 (14.3)	63 (11.5)	
*Sex*						*p* = .591*
Male	148 (55.4)	75 (57.7)	63 (59.4)	21 (47.7)	307 (56.1)	
Female	119 (44.6)	55 (42.3)	43 (40.6)	23 (52.3)	240 (43.9)	
Age (Average and SD)	60.2 (26.1)	44.3 (23.1)	69.6 (18.7)	72.4 (17.9)	59.2 (25.3)	*p* < .001**
*Age groups*						
0–17 year	26 (9.7)	11 (8.5)	2 (1.9)	0 (0.0)	39 (7.1)	
18–34 year	23 (8.6)	41 (31.5)	5 (4.7)	2 (4.5)	71 (13.0)	
35–49 year	21 (7.9)	30 (23.1)	10 (9.4)	6 (13.6)	67 (12.2)	
50–64 year	47 (17.6)	18 (13.8)	15 (14.2)	4 (9.1)	84 (15.4)	
65–84 year	110 (41.2)	27 (20.8)	53 (50.0)	20 (45.5)	210 (38.4)	
85 + years	40 (15.0)	3 (2.3)	21 (19.8)	12 (27.3)	76 (13.9)	
*Day of the week*						*p* = 0.246*
Monday	52 (19.5)	16 (12.3)	14 (13.2)	10 (22.7)	92 (16.8)	
Tuesday	33 (12.4)	19 (14.6)	19 (17.9)	8 (18.2)	79 (14.4)	
Wednesday	32 (12.0)	18 (13.8)	13 (12.3)	7 (15.9)	70 (12.8)	
Thursday	32 (12.0)	14 (10.8)	20 (18.9)	2 (5.5)	68 (12.4)	
Friday	46 (17.2)	29 (22.3)	20 (18.9)	9 (20.5)	104 (19.0)	
Saturday	34 (12.7)	18 (13.8)	8 (7.5)	7 (15.9)	67 (12.2)	
Sunday	38 (14.2)	16 (12.3)	12 (11.3)	1 (2.3)	67 (12.2)	
*Applicant*						*p* < .001*
General practitioner	81 (30.3)	13 (10.0)	78 (73.6)	13 (29.5)	185 (33.8)	
112 emergency number	93 (34.8)	64 (49.2)	5 (4.7)	5 (11.4)	167 (30.5)	
Out-of-hours general practitioner	18 (6.7)	11 (8.5)	7 (6.6)	9 (20.5)	45 (8.2)	
Healthcare institute	9 (3.4)	2 (1.5)	5 (4.7)	6 (13.6)	22 (4.0)	
Police	1 (0.4)	10 (7.7)	0 (0.0)	0 (0.0)	11 (2.0)	
Psychiatrist	3 (1.1)	6 (4.6)	2 (1.9)	0 (0.0)	11 (2.0)	
Midwife	1 (0.4)	0 (0.0)	0 (0.0)	1 (2.3)	2 (0.4)	
Other	1 (0.4)	1 (0.8)	1 (0.9)	0 (0.0)	3 (0.6)	
Fire department	1 (0.4)	0 (0.0)	0 (0.0)	0 (0.0)	1 (0.2)	
Unregistered	59 (22.1)	23 (17.7)	8 (7.5)	10 (22.7)	100 (18.3)	
*Dispatch urgency*						*p* < .001*
A1	159 (59.6)	73 (56.2)	17 (16.0)	20 (45.5)	269 (49.2)	
A2	82 (30.7)	52 (40.0)	56 (52.8)	15 (34.1)	205 (37.5)	
B	26 (9.7)	5 (3.8)	33 (31.3)	9 (20.5)	73 (13.3)	
*Medical specialism*						*p* < .001*
Internal medicine	75 (28.1)	43 (33.1)	33 (31.1)	15 (34.1)	166 (30.3)	
Neurology	52 (19.5)	12 (9.2)	10 (9.4)	1 (2.3)	75 (13.7)	
Cardiology	32 (12.0)	5 (3.8)	14 (13.2)	9 (20.5)	60 (11.0)	
Psychiatry	5 (1.9)	39 (30.0)	6 (5.7)	1 (2.3)	51 (9.3)	
Pulmonology	31 (11.6)	6 (4.6)	12 (11.3)	2 (4.5)	51 (9.3)	
Traumatology	12 (4.5)	5 (3.8)	6 (5.7)	2 (4.5)	25 (4.6)	
Pediatry	16 (6.0)	2 (1.5)	0 (0.0)	0 (0.0)	18 (3.3)	
Gynaecology	1 (0.4)	0 (0.0)	0 (0.0)	1 (2.3)	2 (0.4)	
Unregistered	43 (16.1)	18 (13.8)	25 (23.6)	13 (29.5)	99 (18.1)	
*Outcome*						*p* = < .001*
Conveyance	218 (81.6)	48 (36.9)	105 (99.1)	32 (72.3)	403 (73.7)	
Non-conveyance	49 (18.4)	82 (63.1)	1 (0.9)	12 (23.7)	144 (26.3)	

^*^Chi-square test

^**^Kruskal–Wallis test

### Patient characteristics

As for patient characteristics, men (56.1%) were more present in the total re-contact group compared to women (43.9%). This distribution was also present in the groups with re-contact related to illness, the patient, and the professional. Women were more present in the unrelated group compared to men (52.3% vs. 47.7%). χ^2^ showed no significant association between sex and the main categories (Pearson 1,913, *p* = 0.591). The average age for patients with a re-contact was 59.2 years (standard deviation 25.3 years). There was a significant difference in age between the main categories (Kruskal–Wallis *H*(2) = 66,587, *p* < 0.001), were patients in the professional related and unrelated categories were older. 286/547 (52.3%) of the patients with a re-contact was older than 65 years. Re-contacts occurred on all days of the week, with no association between weekday and main categories. 64.3% of the re-contacts were requested by the GP (33.8%) or through the 112 emergency number (30.5%), with a significant association between main categories and the type of applicant. 49.2% of the re-contacts were dispatched with the highest urgency level. There was a significant difference in urgency levels between the main categories, with the professional related category having the most planned re-contacts (Cramer’s V 0,287, *p* < 0.001). There was a significant association between the medical specialism and the main categories (Cramer’s V 0,265, *p* < 0.001). The top-3 medical specialisms accounts for 55.0% of the re-contacts and consisted of internal medicine (30.3%), neurology (13.7%), and cardiology (11.0%). This top-3 is reflected in the illness related category. Psychiatry represented 30.0% of the medical specialisms in the patient related group.

### Subreasons for re-contacts

Subcategories are presented in Table 3. For illness related re-contacts, progression of disease (39.7%) and recurrent disease process/exacerbation (38.2%) were the most common subcategories. Within the patient related re-contacts, 61.5% was related to psychiatric disorder and/or substance abuse. Another 22.3% was related to the patient and/or relatives being worried. Professional related re-contacts were mainly related to reassessment and ambulance request by another medical/care professional (82.1%). Misdiagnoses (10.4%) and treatment errors (7.5%) accounted for a small part within this main category. Across the main categories, four subcategories accounted for 68.5% of all reasons for re-contacts: progression of disease (19.4%), recurrent disease process/exacerbation (18.6%), reassessment and ambulance request by another medical professional (15.9%), and psychiatric disorder and/or substance abuse (14.6%).

**Table 3:** Main and subcategories (n = 547)

	N	Within main category (%)	Total (%)
*Illness related*	267		48.8
Progression of disease	106	39.7	19.4
Recurrent disease process/exacerbation	102	38.2	18.6
Related disease	48	18.0	8.8
Complication	6	2.2	1.1
Additional diagnostics performed, no change in diagnosis	5	1.9	0.9
*Patient related*	130		23.8
Psychiatric disorder and/or substance abuse	80	61.5	14.6
The patient and/or relatives are worried	29	22.3	5.3
Refusal of medical care/treatment	16	12.3	2.9
Non-compliance with self-care instructions	4	3.1	0.7
Non-compliance with instructions to visit own general practitioner	1	0.8	0.2
*Professional related*	106		19.4
Reassessment and ambulance request by another medical/care professional	87	82.1	15.9
Misdiagnosis	11	10.4	2.0
Treatment error	8	7.5	1.5
*Unrelated*	44	100	8.0

### Working diagnosis

Within the subcategory ‘progression of disease’ (n = 106), the top-3 working diagnoses were pneumonia (9.4%), infectious disease (8.5%), and unspecified (5.6%). For the subcategory ‘recurrent disease process/exacerbation’ (n = 102) epileptic convulsions (20.6%), fever convulsions (9.8%), and unspecified (5.9%) accounted for the top-3 working diagnosis. For ‘reassessment and ambulance request by another medical professional’ (n = 87) the top-3 consisted of unspecified (13.8%), angina pectoris (9.2%), and general malaise (8.0%). Within the subcategory ‘psychiatric disorder and/or substance abuse’ (n = 80), panic attack (18.8%), unspecified (18.8%), and intoxication (16.3%) completed the top-3 working diagnosis.

### Outcomes

In the total population, 403/547 (73.7%) patients with a re-contact were conveyed to the hospital. Patients with a re-contact related to illness, the professional or unrelated were mostly conveyed to the hospital. Within the patient related category, 63.1% of the patients were not conveyed. χ^2^ showed a significant association between the main categories and outcome (Cramer’s V 0.496, *p* < 0.001). Mortality rate for re-contacts was 0.5% (3/585): two patients were resuscitated during the re-contact, one patient was found dead on-scene.

## Discussion

This study provides a first insight into reasons for ambulance re-contacts after non-conveyance. Our results show a total re-contact incidence rate of 4.2% within 3 days after the initial non-conveyance, with respective incidence rates for the 0-24 h, 24-48 h and 48-72 h timeframes of 2.8%, 0.9% and 0.5%. Previous studies reported re-contacts rates of 6.1–6.3% (0 h–24 h), 2.3–5.6% (24 h–48 h), and 9.0–20.8% (0 h–72 h) [8, 17–21]. Compared to these studies, the re-contact rates in our study are low. Within these re-contacts, only a small proportion (3.5%) is related to a wrong diagnosis or treatment error. This is low compared to a similar study in the ED-setting, that reported 7.4% misdiagnosis and treatment errors as reasons for unplanned ED revisits [16]. From patient safety perspective our results indicate safe non-conveyance decisions.

A main finding of our study is that within the quality indicator of ambulance re-contacts, a wide variety of reasons underly why patients re-contact ambulance care. This questions the validity of solely using the percentage of re-contacts as quality indicator for non-conveyance. Specifying the quality indicator into categories related to illness, the patient, professionals or even unrelated re-contacts, does more justice to the complex reality of non-conveyance. In addition to measuring re-contacts, indicators within the chain of emergency care should be developed and implemented to gain insight in non-conveyance safety and quality [10].

In our population almost half of ambulance re-contacts are related to illness, with a large proportion of re-contacts due to progression of disease and recurrent disease process/exacerbation. Similar studies in ED-settings report comparable results for unplanned ED visits [16, 22]. The high proportion of illness related re-contacts is in line with a previous study that reported that repeated use of ambulances was associated with chronic health problems and comorbidities [1]. An ‘unspecified working diagnosis’ is part of the top-3 working diagnosis of all of the four most common subcategories. Within the context of non-conveyance decision-making these results indicate room for the (further) development of clinical reasoning of ambulance care professionals. Previous studies described that clinical reasoning and decision-making in non-conveyance situations is experienced as complex and complicated by ambulance care professionals [23–25]. So besides more education and training on non-conveyance clinical reasoning and decision making, point-of-care tests and tools with predictive value for disease progression should be developed and implemented. Point-of-care tests in acute care (such as troponin, CRP or ultrasound) and risk stratification tools, support the diagnostic process and clinical decision-making, for example for whether or not to convey the patient [15, 26–28]. In addition, there is room for the development of tools with predictive value for disease progression, patient outcomes and re-contacts for non-conveyed patients. NEWS2 scores have been associated with re-contacts mortality in non-conveyed patients, with higher NEWS2 scores indicating a higher likelihood of re-contacts or death [2, 19, 21]. In addition to clinical reasoning, shared decision making could be valuable to the patient group the re-contacts the ambulance due to worrying, in our study 5.3%. Patients calling an ambulance due to worrying, regardless medical urgency, is described earlier [29].

The mortality rate in this study population was 0.5%. Previous studies reported 0.1–0.7% mortality rates at re-contact after non-conveyance [17, 19, 20]. Almost three-quarters (73.7%) of patients were conveyed as outcome of the re-contact. A previous study reported 80.1% conveyance at re-contact [20]. Our results showed a correlation between the main categories of reason and the re-contact. Within the patient related category, 63.1% of the patients were not conveyed. A possible explanation is that this main category consists of sub-reasons with patient refusal, non-compliance to (self-care) advice, and patients and/or relatives being worried. The patient related category also includes the group of patients for whom a psychiatric disorder/substance use is the main reason for the re-contact. Previous studies reported that this patient group is a frequent user of ambulance care, has a higher change of being non-conveyed, more often discharge themselves from care, and have a higher rate of re-contacts [9, 30, 31] For this patient group, this urges the need to develop alternative care pathways.

The framework from this study might provide a valuable tool for EMSs to refine their re-contact indicators. The initial framework was developed for, and used in ED settings. By adding one main category (unrelated) and a few subcategories, the framework was applicable to asses 93.5% of the re-contacts in our study. Using a framework within the EMS setting that is similar to the ED settings enhances comparability within the chain of emergency care. To increase validity and reliability, the framework should be applied in different EMSs at the same time.

### Limitations

The first limitation is the retrospective design and missing information. Due to suboptimal registration within the medical records, 6.5% of the re-contacts could not be categorized. Secondly, our study did not incorporate follow-up care at the ED or GP for patients who were conveyed at the re-contact ambulance run. Thirdly, all assessors are employed at the EMS were the study was conducted. Although they were blinded, they might have assessed their own ambulance runs. Finally, this study had a single center character as it was conducted at one EMS region in the Dutch EMS-system, possibly limiting generalizability to other EMSs in the Netherlands or other EMS-systems. EMSs in the Netherlands cover different areas with different populations, geography, triage systems, urbanization grades, and hospital coverage. Also, composition of EMS workforces are slightly different between Dutch EMSs, due to varying ratios between nurses, bachelors of health, nurse practitioners and physician assistants.

## Conclusion

This study shows low re-contact after ambulance non-conveyance. A very small part of re-contacts is related to ambulance care professionals making errors in diagnosis or treatment and mortality at re-contacts was low. This indicates safe non-conveyance decisions. Re-contacts as quality indicator cover a variety of reasons, with almost half of the re-contacts being related to illness. Four subcategories account for the majority of all reasons for re-contacts: progression of disease, recurrent disease process/exacerbation, reassessment and ambulance request by another medical professional, and psychiatric disorder and/or substance abuse. Three-quarters of the patients are conveyed, although more re-contact due to patient related reasons end in non-conveyance again.

## Data Availability

The datasets generated and/or analyzed during the current study are not publicly available as it contains parts of ambulance medical records, but are available from the corresponding author on reasonable request.

## Abbreviations

BH: Bachelor of Health
ED: Emergency department
EMS: Emergency medical service
GP: General practitioner
NEWS: National early warning score

## References

[1] Søvsø MB, Kløjgaard TA, Hansen PA, Christensen EF. Repeated ambulance use is associated with chronic diseases—a population-based historic cohort study of patients’ symptoms and diagnoses. Scand J Trauma Resusc Emerg Med. 2019;27:46. 30992042 10.1186/s13049-019-0624-4 PMC6469091

[2] Heinonen K, Puolakka T, Salmi H, Boyd J, Laiho M, Porthan K, et al. Ambulance crew-initiated non-conveyance in the Helsinki EMS system-a retrospective cohort study. Acta Anaesthesiol Scand. 2022;66:625–33. 35170028 10.1111/aas.14049 PMC9544076

[3] Pittet V, Burnand B, Yersin B, Carron P-N. Trends of pre-hospital emergency medical services activity over 10 years: a population-based registry analysis. BMC Health Serv Res. 2014;14:380. 25209450 10.1186/1472-6963-14-380 PMC4169798

[4] Booker MJ, Shaw ARG, Purdy S. Why do patients with “primary care sensitive” problems access ambulance services? A systematic mapping review of the literature. BMJ Open. 2015;5: e007726. 25991458 10.1136/bmjopen-2015-007726 PMC4442240

[5] Maninchedda M, Proia AS, Bianco L, Aromatario M, Orsi GB, Napoli C. Main features and control strategies to reduce overcrowding in emergency departments: a systematic review of the literature. Risk Manag Healthc Policy. 2023;16:255–66. 36852330 10.2147/RMHP.S399045 PMC9961148

[6] Ebben RHA, Castelijns M, Frenken J, Vloet LCM. Characteristics of non-conveyance ambulance runs: a retrospective study in the Netherlands. World J Emerg Med. 2019;10:239–43. 31534599 10.5847/wjem.j.1920-8642.2019.04.008 PMC6732166

[7] Nederland A. Sectorkompas ambulancezorg [Internet]. Zwolle; 2023. Available from: www.ambulancezorg.nl

[8] Ebben RHA, Vloet LCM, Speijers RF, Tönjes NW, Loef J, Pelgrim T, et al. A patient-safety and professional perspective on non-conveyance in ambulance care: a systematic review. Scand J Trauma Resusc Emerg Med. 2017;25:71. 28716132 10.1186/s13049-017-0409-6 PMC5513207

[9] Lederman J, Lindström V, Elmqvist C, Löfvenmark C, Djärv T. Non-conveyance in the ambulance service: a population-based cohort study in Stockholm. Sweden BMJ Open. 2020;10: e036659. 32665389 10.1136/bmjopen-2019-036659 PMC7365423

[10] Höglund E, Magnusson C, Lederman J, Spangler D, Vloet L, Ebben R. Ambulance quality and outcome measures for general non-conveyed populations (AQUA): a scoping review. PLoS ONE. 2024;19: e0306341. 39163307 10.1371/journal.pone.0306341 PMC11335110

[11] O’Cathain A, Knowles E, Bishop-Edwards L, Coster J, Crum A, Jacques R, et al. Understanding variation in ambulance service non-conveyance rates: a mixed methods study. UK: NIHR Journals Library; 2018. 29870196

[12] von Elm E, Altman DG, Egger M, Pocock SJ, Gøtzsche PC, Vandenbroucke JP. The strengthening the reporting of observational studies in epidemiology (STROBE) statement: guidelines for reporting observational studies. J Clin Epidemiol. 2008;61:344–9. 18313558 10.1016/j.jclinepi.2007.11.008

[13] Dercksen B, Struys MMRF, Cnossen F, Paans W. Qualitative development and content validation of the “SPART” model; a focused ethnography study of observable diagnostic and therapeutic activities in the emergency medical services care process. BMC Emerg Med. 2021;21:135. 34773982 10.1186/s12873-021-00526-z PMC8590330

[14] Loef J, Vloet LCM, Vierhoven P-H, van der Schans L, Neyman-Lubbers Y, de Vries-de WC, et al. Starting ambulance care professionals and critical incidents: a qualitative study on experiences, consequences and coping strategies. BMC Emerg Med. 2021;21:110. 34620095 10.1186/s12873-021-00500-9 PMC8495434

[15] Ort BBA, Uit Het Broek LG, de Bruin H, Akkermans RP, Goosselink B, Vermeulen H, et al. Patient factors associated with conveyance decision-making by Emergency Medical Services professionals in patients with a syncope: a cross-sectional factorial survey design. BMC Emerg Med. 2023;23:118. 37798716 10.1186/s12873-023-00890-y PMC10557231

[16] van der Linden MC, Lindeboom R, de Haan R, van der Linden N, de Deckere ER, Lucas C, et al. Unscheduled return visits to a Dutch inner-city emergency department. Int J Emerg Med. 2014;7:23. 25045407 10.1186/s12245-014-0023-6 PMC4100563

[17] Coster J, O’Cathain A, Jacques R, Crum A, Siriwardena AN, Turner J. Outcomes for patients who contact the emergency ambulance service and are not transported to the emergency department: a data linkage study. Prehospital Emerg care. 2019;23:566–77. 10.1080/10903127.2018.1549628 30582719

[18] Forsell L, Forsberg A, Kisch A, Rantala A. Inequalities and short-term outcome among patients assessed as non-urgent in a Swedish ambulance service setting. Int Emerg Nurs. 2021;57: 101018. 34147876 10.1016/j.ienj.2021.101018

[19] Paulin J, Kurola J, Koivisto M, Iirola T. EMS non-conveyance: a safe practice to decrease ED crowding or a threat to patient safety? BMC Emerg Med. 2021;21: 115. 10.1186/s12873-021-00508-1 PMC8502399 34627138

[20] Todd VF, Swain A, Howie G, Tunnage B, Smith T, Dicker B. Factors associated with emergency medical service reattendance in low acuity patients not transported by ambulance. Prehospital Emerg Care. 2021. 10.1080/10903127.2020.1862943. 10.1080/10903127.2020.1862943 33320722

[21] Todd VF, Moylan M, Howie G, Swain A, Brett A, Smith T, et al. Predictive value of the New Zealand Early Warning Score for early mortality in low-acuity patients discharged at scene by paramedics: an observational study. BMJ Open. 2022;12: e058462. 35835524 10.1136/bmjopen-2021-058462 PMC9289032

[22] Hu K-W, Lu Y-H, Lin H-J, Guo H-R, Foo N-P. Unscheduled return visits with and without admission post emergency department discharge. J Emerg Med. 2012;43:1110–8. 22674038 10.1016/j.jemermed.2012.01.062

[23] Granlund L, Brännström I, Lindström V. Factors influencing non-conveyance care encounters in the ambulance service, registered nurses experiences—a qualitative study. BMC Nurs. 2024;23:271. 38658953 10.1186/s12912-024-01899-9 PMC11044363

[24] Höglund E, Schröder A, Möller M, Andersson-Hagiwara M, Ohlsson-Nevo E. The ambulance nurse experiences of non-conveying patients. J Clin Nurs. 2019;28:235–44. 30016570 10.1111/jocn.14626 PMC8045551

[25] Porter A, Snooks H, Youren A, Gaze S, Whitfield R, Rapport F, et al. “Should I stay or should I go?” Deciding whether to go to hospital after a 999 call. J Health Serv Res Policy. 2007;12(Suppl 1):32–8. 10.1258/135581907780318392 17411505

[26] Tolsma RT, de Koning ER, Fokkert MJ, van der Waarden NW, van ’t Hof AW, Backus BE. Management of patients suspected for non-ST elevation-acute coronary syndrome in the prehospital phase. Future Cardiol. 2023;19:639–47. 10.2217/fca-2023-0049 37916603

[27] Goyder C, Tan PS, Verbakel J, Ananthakumar T, Lee JJ, Hayward G, et al. Impact of point-of-care panel tests in ambulatory care: a systematic review and meta-analysis. BMJ Open. 2020;10: e032132. 32111610 10.1136/bmjopen-2019-032132 PMC7050348

[28] Bøtker MT, Jacobsen L, Rudolph SS, Knudsen L. The role of point of care ultrasound in prehospital critical care: a systematic review. Scand J Trauma Resusc Emerg Med. 2018;26:51. 29940990 10.1186/s13049-018-0518-x PMC6019293

[29] Edwards MJ, Bassett G, Sinden L, Fothergill RT. Frequent callers to the ambulance service: patient profiling and impact of case management on patient utilisation of the ambulance service. Emerg Med J. 2015;32:392–6. 25312857 10.1136/emermed-2013-203496

[30] Duncan EAS, Best C, Dougall N, Skar S, Evans J, Corfield AR, et al. Epidemiology of emergency ambulance service calls related to mental health problems and self harm: a national record linkage study. Scand J Trauma Resusc Emerg Med. 2019;27:34. 30894214 10.1186/s13049-019-0611-9 PMC6425659

[31] Andrew E, Nehme Z, Bernard S, Cameron P, Smith K. 24 Factors associated with emergency ambulance re-attendance within 24 hours of being discharged at scene. BMJ Open. 2019;9:A9–A9.

